# Exciton Delocalization and Polarizability in Perylenetetracarboxylic Diimide Probed Using Electroabsorption and Fluorescence Spectroscopies

**DOI:** 10.3390/molecules29102206

**Published:** 2024-05-08

**Authors:** Md. Bulu Rahman, Ahatashamul Islam, Toshifumi Iimori

**Affiliations:** Department of Sciences and Informatics, Muroran Institute of Technology, Mizumoto-cho 27-1, Muroran 050-8585, Hokkaido, Japan; 22096511@muroran-it.ac.jp (M.B.R.); ahatashamul.islam.264@gmail.com (A.I.)

**Keywords:** n-type organic semiconductors, exciton, self-assembly, electric fields, fluorescence

## Abstract

Perylenetetracarboxylic diimide (PTCDI) is an n-type organic semiconductor molecule that has been widely utilized in numerous applications such as photocatalysis and field-effect transistors. Polarizability and dipole moment, which are inherent properties of molecules, are important parameters that determine their responses to external electric and optical fields, physical properties, and reactivity. These parameters are fundamentally important for the design of innovative materials. In this study, the effects of external electric fields on absorption and fluorescence spectra were investigated to obtain the PTCDI parameters. The PTCDI substituted by an octyl group (*N*,*N*′-Dioctyl-3,4,9,10-perylenedicarboximide) dispersed in a polymethyl methacrylate (PMMA) matrix was studied in this work. The features of vibronic progression in the absorption spectrum were analogous to those observed in solution. The red shift of the absorption band caused by the Stark effect was mainly observed in the presence of an external electric field. Changes in parameters such as the dipole moment and polarizability between the ground and the Franck–Condon excited states of the PTCDI monomer were determined. The fluorescence spectrum shows a contribution from a broad fluorescence band at wavelengths longer than the monomer fluorescence band. This broad fluorescence is ascribed to the excimer-like fluorescence of PTCDI. The effects of the electric field on the fluorescence spectrum, known as the Stark fluorescence or electrofluorescence spectrum, were measured. Fluorescence quenching is observed in the presence of an external electric field. The change in the polarizability of the monomer fluorescence band is in good agreement with that of the electroabsorption spectrum. A larger change in the polarizability was observed for the excimer-like fluorescence band than that for the monomer band. This result is consistent with exciton delocalization between PTCDI molecules in the excimer-like state.

## 1. Introduction

Currently, π-conjugated organic semiconductors have found widespread application in numerous optoelectronic devices [1,2,3,4,5], often substituting traditional inorganic semiconductors. Organic materials have benefits over inorganic materials owing to their reduced toxicity, their technological advantages for creating photoconductive compositions, and the simplicity of manufacturing large-scale devices [6,7,8]. Organic semiconductors have been extensively used in a broad range of optoelectronic and photonic applications. Among these, perylenetetracarboxylic diimide (PTCDI) derivatives have been employed in various fields such as photovoltaics [9,10], biosensors [11,12,13,14], photocatalysis [15,16,17], and energy storage [18,19]. PTCDIs have garnered considerable attention among organic semiconductors because of their low cost, high molar absorption coefficients ranging from 10^4^ to 10^5^ mol^−1^ L cm^−1^, superior ability to emit fluorescence with a high quantum yield (QY), and remarkable resistance to heat and light degradation [6,20]. They exhibit n-type organic semiconductor features and have become a focal point for the development of optoelectronic devices, organic thin-film transistors, sensors, organic solar cells, and nanowires/nanofibers [21,22,23,24,25]. Their optoelectronic properties can be altered by connecting various functional compounds to distinct molecular sites [26].

**Figure 1:**
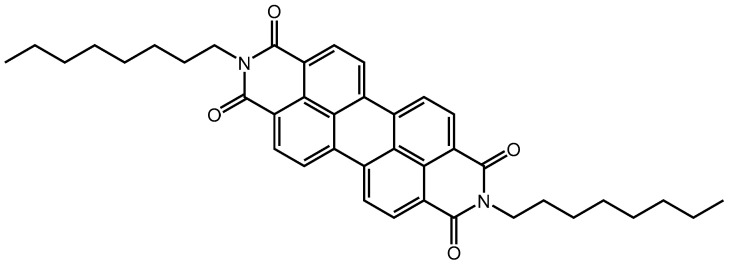
Molecular structure of PTCDI-C_8_.

The physical and chemical properties can be controlled by changing the substituents on the imide N atom. PTCDI substituted with the octyl group PTCDI-C_8_ (*N*,*N*′-Dioctyl 3,4,9,10-perylenedicarboximide) (Figure 1) has been used as a favorable material for diverse applications in organic electronics [27]. PTCDI-C_8_ is also attractive for its photosensitivity, ambient stability, and notable electron mobility ranging from 0.6 to 1.7 cm^2^/Vs [28,29,30], which is greater than that of fullerene. It can also form nanostructures such as nanowires and nanoribbons for nanotechnology applications through *π-π* stacking owing to the self-assembly of PTCDI-C_8_ molecules [21,22,31,32,33,34,35].

Significant research has been dedicated to investigating the absorption and emission spectra of PTCDI derivatives using UV–Vis spectroscopy and quantum chemical methods [3,4,5,36,37,38,39]. The change in the spectra upon the formation of the aggregates of PTCDI-C_8_ was investigated in different solvents [40]. The influence of the crystalline packing geometry on the extent of the delocalization of the excitonic states among the PTCDI molecules was studied using transient absorption and electroabsorption spectroscopy [29]. The PTCDI molecules forming a slip-stacked geometry exhibited the greatest excitonic delocalization.

The study of the effect of an external electric field on the absorption and fluorescence spectra, known as the Stark effect, has been widely applied as a valuable approach to exploring electronic structures and photoexcitation dynamics [41,42,43]. The energy level of a molecule can be altered depending on the magnitude of the external electric field, and this change is determined by molecular characteristics such as permanent dipole moment and polarizability. Using Stark spectroscopy, it is possible to obtain changes in the electric dipole moment and polarizability of the excited and ground states. The evaluation of the electric dipole moment and polarizability is vital for understanding the electronic structure of molecules in the excited state. These parameters are related to the electrical and optical properties of materials; therefore, the evaluation of these parameters is fundamentally important for their application in organic electronic and optical materials. Furthermore, the molecular parameters acquired through these experimental methods can serve as reference points for validating the accuracy of the results achieved from quantum chemical calculations concerning molecular orbitals and electronic states.

Herein, we report the electroabsorption (EA) and Stark fluorescence (SF) spectra of PTCDI-C_8_ dispersed in a polymer matrix. Using this technique, we measured the difference spectra obtained from the absorption or fluorescence spectra in the presence and absence of external electric fields. EA and SF spectroscopy provide insights into the photophysics, photochemistry, and mechanisms underlying the impacts of electric fields on molecules. In addition, changes in the dipole moments and polarizabilities between the ground and excited states are reported. Fluorescence quenching is observed in the presence of an external field.

## 2. Theoretical Background

When a molecule is subjected to an external electric filed F, the energy level is shifted, and this shifting depends on the electric dipole moment (μ) and the polarizability of the molecule (α). This phenomenon is referred to as the Stark shift. The change in optical transition energy can be represented as $∆E=-∆\mu \cdot F-\frac{1}{2}F\cdot ∆\alpha \cdot F$. This shift depends on the electric dipole moment difference (∆μ) between the ground state (g) and excited state (e), as well as the difference in the polarizability (∆α), which are represented by

(1) $$∆\mu =\mu_{e}-\mu_{g},$$

(2) $$∆\alpha =\alpha_{e}-\alpha_{g},$$

where $\mu_{e}$ is the electric dipole moment of the excited state, $\alpha_{e}$ is the polarizability tensor of the excited state, $\mu_{g}$ is the electric dipole moment of the ground state, and $\alpha_{g}$ is the polarizability tensor of the ground state.

The changes in the absorbance and photoluminescence intensity resulting from the Stark shift were examined using Stark spectroscopy. The difference between the absorption spectra obtained in the presence and absence of external electric fields is referred to as the EA spectrum or Stark absorption spectrum. In the case of photoluminescence or fluorescence spectra, the difference between the spectra in the presence and absence of external electric fields is called the electrophotoluminescence spectrum, or alternatively, the SF spectrum.

The EA spectrum ∆*A* is represented by Liptay’s formula, and it can be expressed as a combination of the original spectrum’s zeroth, first, and second derivatives [44,45,46,47]:

(3) $$∆A\left(\nu \right)={\left(fF\right)}^{2}\left[A_{\chi }A\left(\nu \right)+B_{\chi }\nu \frac{d}{d\nu }\left\{\frac{A\left(\nu \right)}{\nu }\right\}+C_{\chi }\nu \frac{d^{2}}{d\nu^{2}}\left\{\frac{A\left(\nu \right)}{\nu }\right\}\right],$$

where the internal field factor is represented by *f*, *ν* represents the frequency of light, and *F* represents the magnitude of electric field strength applied externally upon the film. *χ* represents the angle formed between the direction of the light’s electric field and the direction of the vector F. The coefficient *A_χ_* corresponds to the contribution of the zeroth derivative, *B_χ_* corresponds to the contribution of the first derivative, and *C_χ_* corresponds to the contribution of the second derivative of the original absorption spectrum *A*(*ν*) divided by *ν*.

In the PMMA matrix, we assumed that the orientation of the molecules in the ensemble was random. Moreover, the reorientation of the molecules in the presence of external electric fields is negligible. The molecules are fixed or immobilized within a rigid polymer matrix. Under these assumptions, the coefficients *A_χ_*, *B_χ_*, and *C_χ_* can be represented by the following equations [41,48]:

(4) $$A_{\chi }=a_{1},$$

(5) $$B_{\chi }=\frac{∆\bar{\alpha }}{2h}+\frac{1}{10h}(3\cos^{2}\chi -1)(∆\alpha_{m}-∆\bar{\alpha })+b_{1},$$

(6) $$C_{\chi }={\left(∆\mu \right)}^{2}\left\{\frac{5+(3\cos^{2}\chi -1)(3\cos^{2}\eta -1)}{30h^{2}}\right\},$$

where *h* and *η* represent Planck’s constant and the angle formed between ∆μ and the transition dipole moment (*m*), respectively. ∆μ is equivalent to |∆μ|, and $∆\bar{\alpha }$ represents the trace of ∆α, i.e., $∆\bar{\alpha }=\frac{1}{3}Tr(∆\alpha )$.

$∆\alpha_{m}$ indicates the alteration of the components of the polarizability along the direction of *m*, and it is denoted by

(7) $$∆\alpha_{m}=m\cdot ∆\alpha \cdot m/{\left|m\right|}^{2}.$$

In Equations (4) and (5), the terms *a*_1_ and *b*_1_ originate from the effect of the external electric field on m [41,48,49].

Although the theoretical framework for SF spectroscopy is similar to that of EA spectroscopy, there is a distinction in the specific molecular properties that can be extracted from the experiment. The EA spectrum arises from changes in the molecular parameters of the Franck–Condon (FC) excited and ground states. In contrast, the SF spectrum arises from differences in the molecular parameters between the fluorescent state and the FC ground state. The spectral profile of SF [$∆I_{FL}(\nu )$] can be expressed in the following manner [43,44,50]:

(8) $$∆I_{FL}\left(\nu \right)={\left(fF\right)}^{2}\left[A^{\prime }I_{FL}\left(\nu \right)+B^{\prime }\nu^{3}\frac{d}{d\nu }\left\{\frac{I_{FL}\left(\nu \right)}{\nu^{3}}\right\}+C^{\prime }\nu^{3}\frac{d^{2}}{d\nu^{2}}\left\{\frac{I_{FL}\left(\nu \right)}{\nu^{3}}\right\}\right],$$

where $I_{FL}\left(\nu \right)$ represents unperturbed fluorescence spectrum. $A^{\prime }$ indicates the contribution of the zeroth derivative component, $B^{\prime }$ indicates the contribution of the first-derivative component, and $C^{\prime }$ indicates the contribution of the second-derivative component of the fluorescence spectrum divided by $\nu^{3}$.

The component $A^{\prime }$ corresponds to the change in fluorescence intensity caused by the applied field. Therefore, the coefficient $A^{\prime }$ arises from the change in fluorescence quantum yield caused by the external field [48,50]. The explicit expressions for coefficients $B^{\prime }$ and $C^{\prime }$ exhibit similarities with Equations (5) and (6) for the EA spectrum. $B^{\prime }$ is linked to the trace of the change in polarizability ($∆{\bar{\alpha }}_{fl}$), and it can be expressed as $∆{\bar{\alpha }}_{fl}=\frac{1}{3}Tr(\alpha_{fl}-\alpha_{g})$, in which $\alpha_{fl}$ represents the polarizability tensor of the fluorescent state and $\alpha_{g}$ represents the polarizability tensor of the FC ground state. An approximate expression of the coefficient $B^{\prime }$ can be given as follows:

(9) $$B^{\prime }=\frac{∆{\bar{\alpha }}_{fl}}{2h}.$$

In this analysis, the provided parameters, along with the calculations, were not adjusted for the internal field factor, which is denoted as *f*. Factor *f* is the conversion factor from an externally applied electric field to a local electric field that affects the molecules within the dielectric materials. In general, the accepted range for the value of *f* in frozen solvent matrices is between 1.1 and 1.3 [42]. All of the reported values for ∆μ, $∆\bar{\alpha },\;∆\alpha_{m},$ and $∆{\bar{\alpha }}_{fl}$ except those obtained from quantum chemical calculations are represented as ∆μ·f, $∆\bar{\alpha }\cdot f^{2}$, $∆\alpha_{m}\cdot f^{2}$, and $∆{\bar{\alpha }}_{fl}\cdot f^{2}$.

## 3. Results and Discussion

### 3.1. Absorption and Electroabsorption Spectra

**Figure 2:**
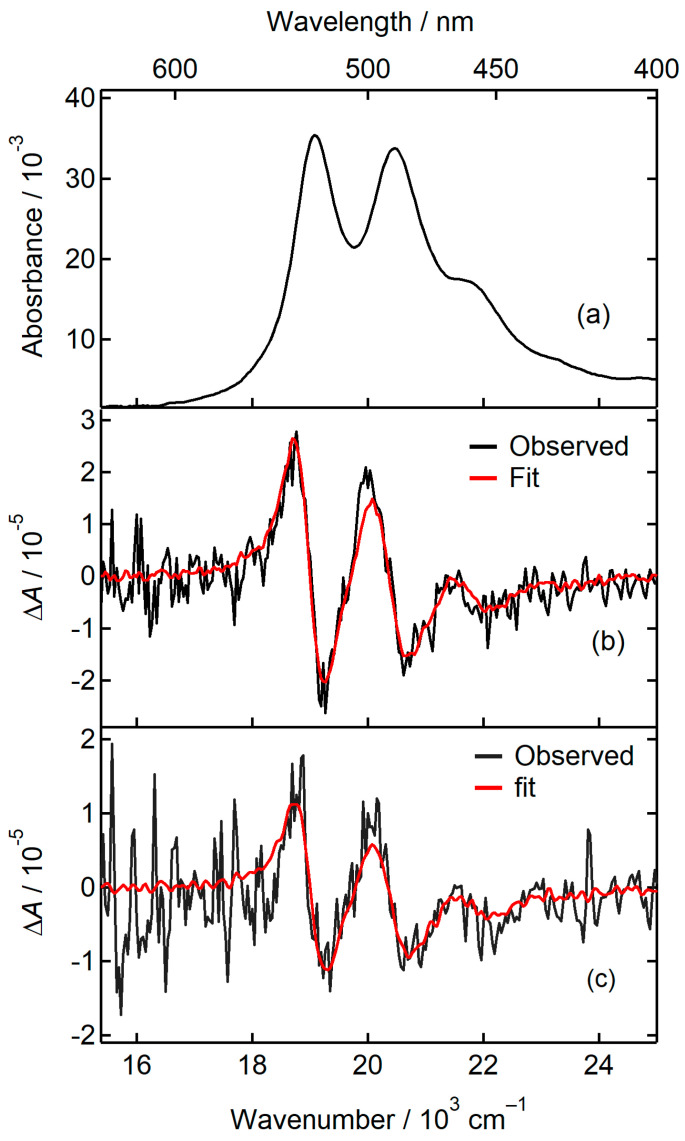
(**a**) Absorption spectrum of PTCDI-C_8_/PMMA of the low concentration. (**b**,**c**) The observed EA spectrum which was obtained at (**b**) *χ* = 55° and (**c**) 90° (black solid lines) and the fitting curve (red solid line).

In Figure 2a, we see the absorption spectra of the PTCDI-C_8_/PMMA of the low concentration. The studied molecule displays a wide absorption band characterized by two well-resolved peaks at approximately 524 nm ($1.9\times 10^{4}\;cm^{-1}$) and 490 nm ($2.04\times 10^{4}\;cm^{-1}$), with a shoulder peak at approximately 460 nm ($2.17\times 10^{4}\;cm^{-1}$). These peaks are assigned to the vibronic band of the S_1_–S_0_ π–π* transition of the monomer of PTCDI-C_8_ [40]. The absorption peaks at 524, 490, and 460 nm correspond to 0–0, 1–0, and 2–0 vibronic transitions of the monomer, respectively. These features are consistent with those reported in previous studies [6,29,31,40].

Figure 2b demonstrates the EA spectrum measured at an angle of χ=55° and electric field strength of $F=0.49\;MV\;cm^{-1}$. The EA spectrum displays an increase in absorbance at wavenumbers below the maximum position of the band. We also noticed a derivative line shape for the two vibronic bands at 499 nm ($2.03\times 10^{4}\;cm^{-1}$) and 533 nm ($1.87\times 10^{4}\;cm^{-1}$), which suggests that owing to the external electric field, a red shift of the absorption band occurs. A similar spectrum was observed at an angle of χ=90° (Figure 2c). We could fit the EA spectrum by employing a single set of coefficients of $A_{\chi }$, $B_{\chi }$, and $C_{\chi }$, as shown in Figure 2b. The fitting coefficients are listed in Table 1.

The zeroth derivative of the absorption component makes an insignificant contribution to the fitting of the EA spectrum in comparison with the first and second-derivative components. This particular component is attributed to the alteration in the transition dipole moment owing to the external electric field, as explained in Equation (4). The shape of the EA spectrum was similar to that of the first derivative of the absorption spectrum. The first-derivative coefficient $B_{\chi }$ (Equation (5)) is formed by combining contributions from three components, such as the polarizability difference $∆\bar{\alpha }$ term, the difference between $∆\alpha_{m}$ and $∆\bar{\alpha }$, and the $b_{1}$ term. In our analysis, we assumed that the contribution of the $b_{1}$ term is negligible. In general, this assumption applies to the optically allowed transitions. A difference was observed in the $B_{\chi }$ values between χ=55° and 90°. The second term in Equation (5) becomes zero at χ=55°. At χ=55°, the only factor that contributes to $B_{\chi }$ is the change in polarizability, which is denoted by $∆\bar{\alpha }$. The magnitude of $∆\bar{\alpha }\cdot f^{2}$ was obtained as 71$\;{Å}^{3}$ (Table 2) from the fitting coefficient of the EA spectrum (Table 1). The observed difference in $B_{\chi }$ between χ=55° and 90° is ascribed to the second term in Equation (5). From the difference in $B_{\chi }$, the magnitude of $(∆\alpha_{m}-∆\bar{\alpha })\cdot f^{2}$ is calculated as 1.2 × 10^2^ ${Å}^{3}$. Subsequently, the estimated value for $∆\alpha_{m}\cdot f^{2}$ is 1.9 × 10^2^ ${Å}^{3}$. The contribution of the second-derivative component is observed in the EA spectrum (Table 1). The change in dipole moment ∆μ is calculated from the coefficient $C_{\chi }$ at an angle of χ=55° (Equation (6)). The value of ∆μ·f is 1.97 D.

The positive value of $∆\bar{\alpha }$ indicates that the polarizability increases in the excited state. This is reasonable because the molecular orbital in the excited state is generally more spatially extended than that in the ground state. The PTCDI core of PTCDI-C_8_ belonged to the D_2h_ point group. The electronic configuration of the S_1_–S_0_ electronic transition arises mainly from the HOMO–LUMO excitation of the PTCDI core, and the symmetry of the S_1_ state is B_1u_ [40]. If the molecular symmetry belongs to the D_2h_ point group, the permanent dipole moment should be zero for both ground and excited states. In this case, we would expect ∆μ = 0 for the S_1_–S_0_ electronic transition of PTCDI-C_8_. However, we obtained a nonzero value for ∆μ. It is inferred that symmetry breaking into a structure with lower symmetry occurs in the excited state.

**Figure 3:**
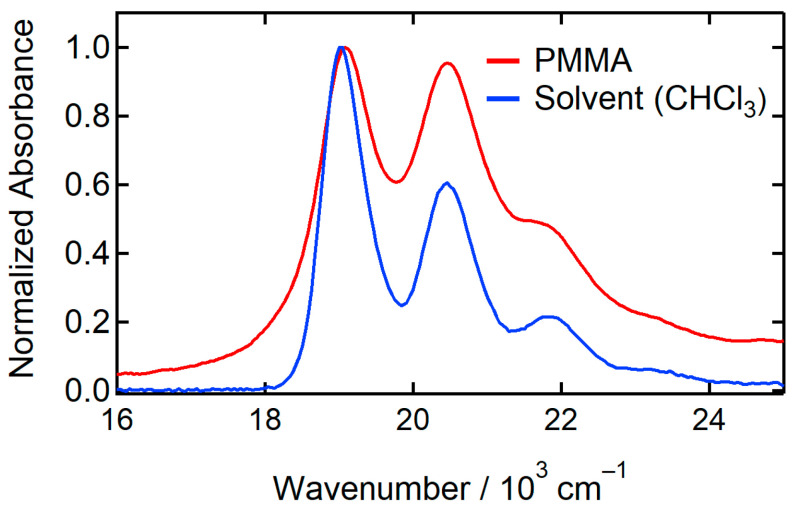
Normalized absorption spectra in PMMA film and chloroform.

The absorption spectrum in PMMA (Figure 2a) has a clear vibrational progression, indicating that most of the molecules are present as a monomer. In Figure 3, we compared the absorption spectra measured in chloroform and PMMA. The bandshape of the absorption spectrum in PMMA is similar to that in solution. This result also shows that the absorption spectrum originates from a monomer.

**Figure 4:**
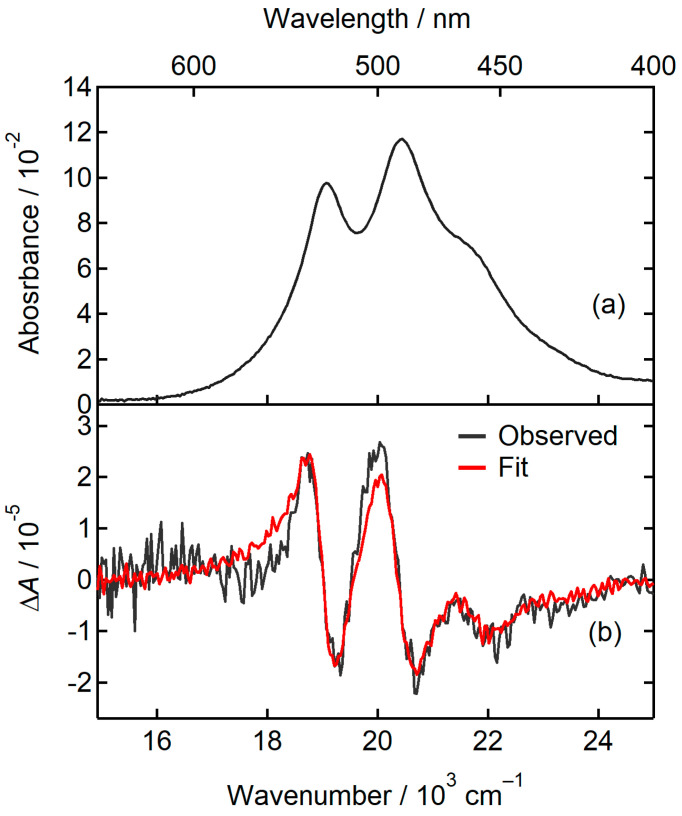
(**a**) Absorption spectrum of PTCDI-C_8_/PMMA of the high concentration. (**b**) The observed EA spectrum obtained at *χ* = 55° (black solid line) and the fitting curve (red solid line).

The absorption and EA spectra of the sample of the high concentration is shown in Figure 4. The bandshape of the absorption spectrum changes from that of the sample of the low concentration. The change is caused by the appearance of a broad absorption band having the maximum at approximately 2.0 × 10^4^ cm^–1^. This absorption band appearing at the high concentration may be ascribed to oligomers such as the dimer of the PTCDI-C_8_. The EA spectrum obtained with $F=0.28\;MV\;cm^{-1}$ was fitted using a single set of the coefficients of $A_{\chi }$, $B_{\chi }$, and $C_{\chi }$ (Figure 4b). If the oligomer of PTCDI-C_8_ had different $∆\bar{\alpha }$ and ∆μ from those of the monomer, the decomposition of the absorption spectrum into the absorption bands of the oligomer and monomer would be necessary to fit the EA spectrum. However, we could fit the EA spectrum using the whole absorption spectrum. These results illustrate that the $∆\bar{\alpha }$ and ∆μ of the oligomer are similar to those for the monomer. The polarizability change $∆\bar{\alpha }\cdot f^{2}=93\;{Å}^{3}$ was calculated from the coefficient $B_{\chi }$ for the fitting in Figure 4b.

(*N*,*N*′-bis(1-pentyl)perylene-3,4,9,10-bis(dicarboximide)) (PTCDI-C_5_) was shown to adopt the slip-stacked packing geometry in the crystal [51]. The difference in molecular structure between PTCDI-C_8_ and PTCDI-C_5_ is only the number of carbon atoms in the alkyl group. Therefore, we assume that the dimer and larger oligomers of PTCDI-C_8_ formed in PMMA film have the slip-stacked geometry. It was reported that the absorption band of the J-aggregate of PTCDI molecules appeared at 592 nm ($1.69\times 10^{4}$ cm^–1^) in DMF or DMSO solutions at high concentration [40]. Whereas the oligomer of PTCDI-C_8_ such as the dimer may be formed in the PTCDI-C_8_/PMMA film, the absorption band of the J-aggregate of PTCDI-C_8_ was negligibly small in Figure 2, Figure 3 and Figure 4. Therefore, the contribution of the J-aggregate to the EA spectrum was negligible.

The values of $∆\bar{\alpha }$ and ∆*μ* were calculated using DFT and TD-DFT calculations (Table 2). The excitation energy and oscillator strength of the first excited singlet state were calculated as 2.44 eV (508 nm) and 0.666, respectively. The PTCDI core of the optimized geometry in the ground state belonged to the D_2h_ point group, and this structure is consistent with the calculation result of ∆*μ* = 0 D. The calculated values of ${\bar{\alpha }}_{g}=\frac{1}{3}Tr(\alpha_{g})$ and ${\bar{\alpha }}_{e}=\frac{1}{3}Tr(\alpha_{e})$ in the ground and excited states were 55 and 66 ${Å}^{3}$, respectively, resulting in $∆\bar{\alpha }=11\;{Å}^{3}$ (Table 2). The DFT calculated value was in the same order of magnitude as that obtained from the analysis of the EA spectrum, whereas a smaller value was obtained from the calculation at this level of theory.

### 3.2. Fluorescence and Stark Fluorescence Spectra

**Figure 5:**
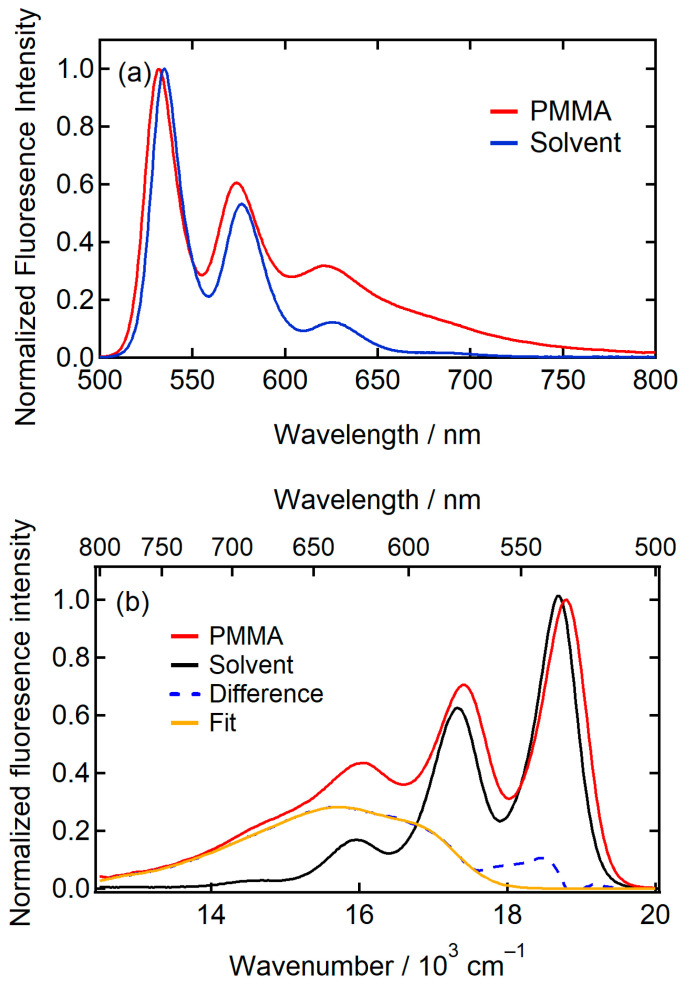
(**a**) Fluorescence spectra in chloroform with a concentration of $1.46\times 10^{-6}$ M (solid blue line) and PMMA (solid red line) obtained with the excitation at 490 nm. The two spectra were normalized at the first vibronic band peak at approximately 535 nm. (**b**) The same spectra with those in (**a**) plotted in the wavenumber scale. (Dashed blue line) The difference spectrum obtained by subtracting the two spectra. (Solid orange line) The fitting result of the excimer-like fluorescence band using a combination of Gaussian bands.

Figure 5 shows the fluorescence spectra of the dilute solution of PTCDI-C_8_ in chloroform and the PMMA film of the low concentration. The concentration of the solution was low enough (1.46 × 10^−6^ M) to observe the monomer fluorescence of PTCDI-C_8_. While the fluorescence spectra display a distinct vibronic structure at approximately 535 and 575 nm, the features of the fluorescence spectra in the solvent and PMMA differ in the region of the longer fluorescence wavelength. This difference originates from the excimer-like fluorescence observed only in PMMA [52]. The excimer-like fluorescence band was extracted by subtracting two spectra shown in Figure 5a. The excimer-like band was derived by subtracting the fluorescence spectrum normalized to the vibronic band observed at 535 nm (Figure 5b). The derived spectrum shows maxima at 635 nm ($1.56\times 10^{4}$ cm^–1^) and 540 nm ($1.85\times 10^{4}$ cm^–1^). The previously reported excimer-like band exhibited a broad bandshape, and the maximum was observed at approximately 640 nm [52]. Therefore, we assigned the broad fluorescence band with a maximum at 635 nm to the excimer-like band in PMMA. Although the origin of the small feature at 540 nm might be a change in the shape of the vibronic band of the monomer fluorescence, we can assume that this feature has a negligible effect on the result obtained from the fitting of the SF spectrum because of the small intensity. The excimer-like fluorescence band was fitted to a combination of Gaussian bands, as shown in Figure 5b. Gaussian band fitting was used to reduce the effect of noise in the fluorescence and derivative spectra. The fitted spectrum was used for further analysis of the SF spectrum.

**Figure 6:**
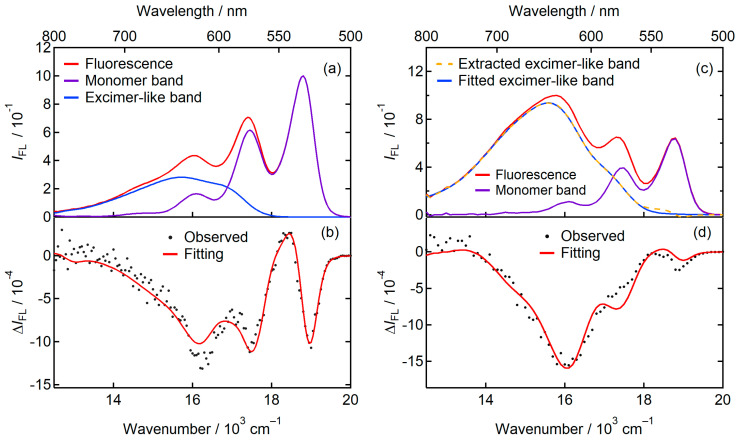
(**a**) (Red line) Fluorescence spectrum measured with excitation at 490 nm, and the decomposition of the fluorescence spectrum into (violet line) the monomer and (blue line) excimer-like fluorescence bands. The excimer-like band fitted using a combination of Gaussian bands in Figure 5 was reproduced. The monomer band was obtained by subtracting the excimer-like band from the fluorescence spectrum. (**b**) (Black dot) The SF spectrum measured at an excitation wavelength of 490 nm, and (red line) the fitting result using the monomer and the excimer-like fluorescence bands. (**c**) (Red line) Fluorescence spectrum measured with excitation at 490 nm. (Violet line) The monomer band taken from (**a**). (Orange dash line) The excimer-like fluorescence band obtained via the subtraction of the monomer band from the fluorescence spectrum. (Blue line) The excimer-like fluorescence band fitted using a combination of Gaussian bands. (**d**) (Black dot) The SF spectrum measured at an excitation wavelength of 490 nm, and (red line) the fitting result using the monomer and the fitted excimer-like fluorescence bands.

Figure 6b demonstrates the SF spectrum of the low concentration sample measured with a field strength of 0.49 MVcm^–1^ with an excitation wavelength of 490 nm, where the EA spectrum exhibits nearly zero amplitude (Figure 2b). This validates that the alteration in absorbance caused by the electric field has minimal effect on the fluorescence intensity. Within the spectral range of SF, a downward signal was observed across all wavelengths. The SF spectrum was fitted by using a set of coefficients $A^{\prime }$, $B^{\prime }$, and $C^{\prime }$ for the monomer band and another set of coefficients for the excimer-like band. (Figure 6b). The coefficients determined by fitting the SF spectrum using the derivatives of the monomer- and excimer-like bands are listed in Table 3.

The fluorescence and SF spectra of the high concentration sample measured with a field strength of $F=0.28\;MV\;cm^{-1}$ are shown in Figure 6c,d. In the fluorescence spectrum, the contribution of the excimer-like fluorescence band increased from that of the low-concentration sample. The excimer-like fluorescence band was extracted from the fluorescence spectrum by subtracting the monomer band from the fluorescence spectrum and further fitted using a combination of Gaussian bands. The monomer band was assumed to be the same with that for the low-concentration sample (Figure 6a). In the fitting of the SF spectrum using Equation (8), the monomer and fitted excimer-like bands were used. The fitting result is shown in Figure 6d.

The zero-derivative coefficients of each band exhibit negative amplitudes. Negative signals denote a reduction in the fluorescence quantum yield upon the application of an external electric field. The first-derivative components influence the SF spectrum along with the zeroth derivative components. From the first-derivative components of each band with the assistance of Equation (9), $∆{\bar{\alpha }}_{fl}$ of the monomer and excimer-like fluorescence bands were calculated (Table 4).

It is seen from Table 2 and Table 4 that the $∆\bar{\alpha }$ and $∆{\bar{\alpha }}_{fl}$ for the monomer absorption and fluorescence bands are in good agreement with each other. The average polarizability change $∆{\bar{\alpha }}_{fl}$ for the excimer-like fluorescence band is larger than $∆\bar{\alpha }$ and $∆{\bar{\alpha }}_{fl}$ for the monomer. It was reported that the $∆{\bar{\alpha }}_{fl}\cdot f^{2}$ of the perylene excimer was 3.4 × 10^2^ Å^3^ [53]. Perylene is the skeleton chromophore of PTCDI-C_8_, and it is reasonable to obtain the similar values of $∆{\bar{\alpha }}_{fl}$ between the perylene excimer and excimer-like PTCDI-C_8_.

Energy transfer is an important process in organic semiconductor molecules such as PTCDI. The energy transfer is coherent when the energy transfer faster than the energy dissipation time occurs. Conversely, when the energy transfer occurs slowly compared to the energy dissipation time, the energy transfer is incoherent, which is also known as Förster-type energy transfer [54]. If *i*-th molecule is in an excited state, ${\left.|EX\right\rangle }_{i}$, and *j*-th molecule is in the ground state, ${\left.|GS\right\rangle }_{j}$, the zeroth-order state with no intermolecular interaction can be represented by the product of these states, ${\left.|EX\right\rangle }_{i}{|\left.GS\right\rangle }_{j}$. The energies of the two zeroth-order electronic configurations represented by ${\left.|EX\right\rangle }_{1}{|\left.GS\right\rangle }_{2}$ and ${|\left.GS\right\rangle }_{1}{\left.|EX\right\rangle }_{2}$ are degenerated in the absence of an electronic coupling between the two molecules. When the coherent energy transfer process is mediated by an electronic coupling, the energy levels of the two electronic configurations are split, which is known as Davidov splitting. The split two electronic states are represented by the linear combination of ${\left.|EX\right\rangle }_{1}{|\left.GS\right\rangle }_{2}$ and ${|\left.GS\right\rangle }_{1}{\left.|EX\right\rangle }_{2}$. The charge transfer interaction also plays an important role in the stabilization of the energy of excimer [55]. The electronic state of an excimer can be represented with the coherent energy transfer and charge transfer between two molecules as follows [54,55]:

(10) $$\Psi =c_{1}\left[{\left.|EX\right\rangle }_{1}{|\left.GS\right\rangle }_{2}\pm {|\left.GS\right\rangle }_{1}{\left.|EX\right\rangle }_{2}\right]+c_{2}\left[{|\left.+\right\rangle }_{1}{|\left.-\right\rangle }_{2}\pm {|\left.-\right\rangle }_{1}{|\left.+\right\rangle }_{2}\right],\;$$

where ${|\left.+\right\rangle }_{i}$ and ${|\left.-\right\rangle }_{i}$ represent the components where a hole and an electron are localized on the *i*-th molecule, respectively. The *c*_1_ and *c*_2_ are coefficients. According to a textbook of molecular quantum mechanics, the polarizability volume is approximately the volume of the molecule [56]. When multiple molecules are electronically coupled, as represented by Equation (10), the excitation energy is more delocalized among the molecules compared with those in the monomer, and the exciton wave function will spatially expand. A larger polarizability than that of the monomer will result from the delocalization of the excitation energy through the coherent energy transfer and charge transfer. For example, the polarizability of the electronically coupled porphyrins has been studied for covalently linked porphyrin oligomers [57]. An increase in the polarizability in the excited state was observed with the increase in the number of the porphyrin unit. This increase in the polarizability was explained by the delocalization of the weakly bound exciton among the porphyrin molecules.

## 4. Experimental and Calculation Methods

PMMA (Sigma Aldrich, St. Louis, MO, USA; average molecular weight: 120,000) was purified via precipitation using benzene and methanol. PTCDI-C_8_ (0.5 or 1.4 mg, Sigma Aldrich) was mixed with 6 mL of chloroform and PMMA (0.8 g) with a magnetic stirrer. After the dissolution of PMMA, the solution of PTCDI-C_8_/PMMA was spin-coated onto a quartz substrate partially coated with a conductive indium tin oxide (ITO) layer (quartz/ITO). The fabricated film was dried overnight in ambient atmosphere. A semi-transparent aluminum layer was deposited on the PTCDI-C_8_/PMMA film via vacuum vapor deposition. To obtain the EA and SF spectra, samples with a layered structure of quartz/ITO/(PTCDI-C_8_/PMMA)/Al were used. ITO and Al films were used as electrodes to apply a high voltage. Hereafter, the sample films prepared using 0.5 and 1.4 mg of PTCDI-C_8_ are called the samples of the low and high concentrations, respectively.

The absorption spectrum of PTCDI-C_8_/PMMA was recorded using a spectrophotometer (Hitachi High-tech, Tokyo, Japan, U4100). The thicknesses of the PTCDI-C_8_/PMMA films were evaluated by analyzing the interference fringes present in the transmission spectra within the near-infrared region. Two films were fabricated, and the average film thickness was approximately 9 and 15 μm for the samples of the low and high concentrations, respectively.

We utilized a custom-made electric field modulation spectroscopy setup to acquire the EA and SF spectra. The detail of the setup was reported elsewhere [50,58]. In brief, an alternating-current (AC) voltage of $f_{0}=333\;Hz$ was applied to the film. The modulation signal of the intensity of the transmitted light or the fluorescence was detected using a lock-in amplifier at the second harmonic, 2*f*_0_. The EA and SF spectra were calculated using the modulation signal amplitude and the direct current component. In SF spectroscopy, unpolarized excitation light was used, and the unpolarized fluorescence spectrum was measured. In EA spectroscopy, the linearly polarized excitation light was used. In the analysis of the EA spectrum, the derivative spectrum of the absorption spectrum was numerically calculated. The derivative spectrum tends to be significantly affected by small noise present in the absorption spectrum. To reduce the deterioration effect of the noise of the derivative spectrum in the fitting of the EA spectrum, the absorption spectrum was smoothed using Savitzky–Golay smoothing.

Density functional theory (DFT) calculations of the PTCDI monomer were performed using Gaussian16 at the B3LYP/6-31G(d,p) level [59]. We used a PTCDI molecule substituted with the methyl group (*N*,*N*′-Dimethyl 3,4,9,10-perylenedicarboximide) instead of PTCDI-C_8_ to reduce the calculation cost. The geometry optimization in the ground state was performed, and then the static polarizability ($\alpha_{g}$) and dipole moment ($\mu_{g}$) were calculated. Time-dependent DFT (TDDFT) calculation using the geometry optimized in the ground state was performed to calculate the $\alpha_{e}$ and $\mu_{e}$ of the first singlet excited state.

## 5. Conclusions

PTCDI-C_8_, an organic semiconductor molecule, has been extensively employed in various fields, such as photovoltaic technology, photocatalysis, and biosensor technology. In this study, the EA and SF spectra of PTCDI-C_8_ in a polymer matrix were measured to investigate its excited-state properties. The changes in the parameters such as the dipole moment and polarizability upon the optical transition in the monomer of PTCDI-C_8_ were evaluated to be 71 $f^{-2}$ ${Å}^{3}$ and 1.97 $f^{-1}$ D, respectively, using EA spectroscopy. By comparing the fluorescence spectra observed in the solvent and the PMMA solid film, a broad fluorescence band with a maximum at approximately 635 nm, which was different from the monomer fluorescence of PTCDI-C_8_, was identified. This fluorescence band was assigned as an excimer-like fluorescence band. The SF spectrum exhibits fluorescence quenching in the presence of an electric field. The SF spectrum was fitted using different sets of fitting coefficients for the monomer and excimer-like fluorescence bands, which enabled individual determination of the parameters for the two molecular species. The change in the polarizability for the excimer-like fluorescence band was obtained as 3.3 × 10^2^ $f^{-2}\;{Å}^{3}$ for the low concentration and 5.5 × 10^2^$\;f^{-2}\;{Å}^{3}$ for the high concentration of PTCDI-C_8_, respectively. The value of the polarizability was larger for the excimer-like fluorescence band than that for the monomer fluorescence band (46 $f^{-2}$ ${Å}^{3}$). This result was consistent with the delocalization of the excitation energy through the coherent energy transfer and charge transfer between molecules in the excimer-like state.

## Figures and Tables

**Table 1:** Coefficients used to fit the EA spectrum of the sample of the low concentration.

χ	$A_{\chi }$$(10^{-4}\;cm^{2}\;MV^{-2}$)	$B_{\chi }$(cm MV^−2^)	$C_{\chi }$(MV^−2^)
55°	−0.9	2.0	180
90°	−3.3	1.3	60

**Table 2:** Parameters of the monomer of PTCDI-C_8_.

Parameter	Experiment ^a^	Calculation ^b^
$∆\bar{\alpha }\cdot f^{2}$	71 ${Å}^{3}$	11 ${Å}^{3}$
∆*μ*·f	1.97 D	0 D

^a^ Values experimentally obtained from the EA spectrum. ^b^ Values obtained from the TD-DFT calculation at the level of B3LYP/6−31G(d,p) for the methyl-substituted PTCDI in gas phase, where *f* is unity. See the text about the detailed molecular structure and the values for $\bar{\alpha }$ in the ground and excited states.

**Table 3:** Coefficients used to fit the SF spectrum of the sample of the low concentration.

	$A^{\prime }$ ($10^{-2}\;cm^{2}\;MV^{-2}$)	$B^{\prime }$ (cm MV^−2^)	$C^{\prime }$ (10^2^ MV^−2^)
Monomer	−0.11	1.3	1
Excimer-like	−1.04	9.3	0

**Table 4:** Parameters obtained from the fitting of the SF spectrum.

Fluorescence Bands	Monomer (Low Concentration) ^a^	Excimer-like (Low Concentration) ^a^	Excimer-like (High Concentration) ^b^
$∆{\bar{\alpha }}_{fl}\cdot f^{2}\;({Å}^{3})$	46	3.3 × 10^2^	5.5 × 10^2^

^a^ Result of the sample of the low concentration. ^b^ Result of the sample of the high concentration.

## Data Availability

The data presented in this study are available on reasonable request from the corresponding author.
